# How vertebrates got their bite

**DOI:** 10.7554/eLife.84118

**Published:** 2022-11-15

**Authors:** Thomas F Schilling, Pierre Le Pabic

**Affiliations:** 1 Department of Developmental and Cell Biology, University of California, Irvine Irvine United States; 2 Department of Biology and Marine Biology, University of North Carolina Wilmington United States

**Keywords:** cis-regulatory element, enhancer deletion, nkx3.2, jaw joint, gnathostome, evolution, Zebrafish

## Abstract

A newly discovered enhancer region may have allowed vertebrates to evolve the ability to open and close their jaws.

**Related research article** Leyhr J, Waldmann L, Filipek-Górniok B, Zhang H, Allalou A, Haitina T. 2022. A novel cis-regulatory element drives early expression of *Nkx3.2* in the gnathostome primary jaw joint. *eLife* **11**:e75749. doi: 10.7554/eLife.75749.

Most vertebrates, including humans, evolved from jawless fish which roamed the oceans 420–390 million years ago (Forey and Janvier, 1993). Acquiring jaws allowed our ancestors to bite and chew, expanding the range of food they could eat and where they could live. Understanding how this mouth structure arose is therefore a central question in evolution (Miyashita, 2016).

Studies in lampreys and hagfish, the only species of jawless fish that still exist today, suggest that the jaw evolved from a pre-existing skeletal system surrounding the mouth and throat that was used for filtering food and breathing. A key step in this process was the acquisition of a mobile joint, essentially a skeletal hinge that can open and close the mouth. For this to happen, cells within the jaw skeleton – most likely cartilage cells – had to alter their gene expression to become more flexible. Such changes often involve enhancers, regions of DNA that control when a nearby gene is expressed, and in which part of the body.

Very few enhancer sequences have been preserved between species over long evolutionary periods (Long et al., 2016), and these often control processes related to development (Pennacchio et al., 2006; Bejerano et al., 2004; Lettice et al., 2003; Kvon et al., 2016; Leal and Cohn, 2016; Letelier et al., 2018). Now, in eLife, Tatjana Haitina and colleagues from Uppsala University – including Jake Leyhr as first author – report the discovery of a widely conserved enhancer named Joint Regulatory Sequence 1 (JRS1) that is critical for the development of the jaw joint and, potentially, the early evolution of the jaw (Leyhr et al., 2022).

The team spotted JRS1 by comparing the genomes of multiple species and noticing a sequence present in most jawed vertebrates, but missing in lampreys and hagfish (Figure 1). Experiments in zebrafish revealed that this enhancer drives the expression of a gene called *nkx3.2,* which encodes a transcription factor essential for jaw joint development. This gene is specifically expressed in the jaw joint of embryos, where it regulates the activity of other genes needed to form the hinge that opens and closes the mouth (Miller et al., 2003; Waldmann et al., 2021; Smeeton et al., 2021).

**Figure 1.**
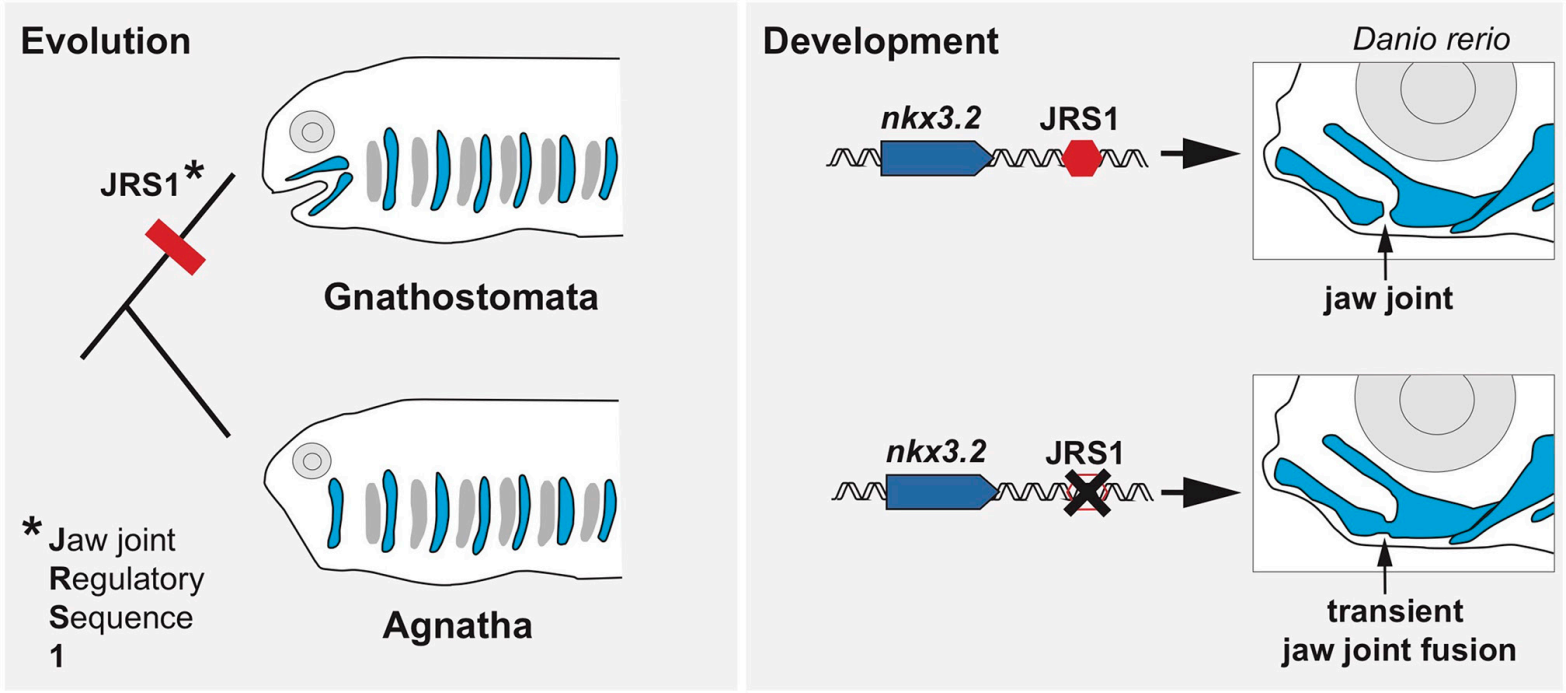
The role of the enhancer JRS1 in evolution and development. Most species in the family of jawed vertebrates (Gnathostomata) evolved from jawless fish (Agnatha). Leyhr et al. have identified an enhancer sequence – which they call JRS1 (short for Jaw joint Regulatory Sequence; left) – that is present in the genomes of multiple species of Gnathostomata, but is absent from the two living Agnathan species (lampreys and hagfish). This genetic sequence (red hexagon) drives expression of the gene *nkx3.2* (blue arrow) in the jaw joint (black arrow) of zebrafish *Danio rerio* embryos (top right). Deleting JRS1 (black cross) eliminates *nxk3.2* expression, leading to jaw joint fusion and the mouth no longer being able to open and close (bottom right). Images of Gnathostomata and Agnatha are based on drawings by Goodrich, 1930.

To investigate where JRS1 drives expression of *nxk3.2* across jawed vertebrates, Leyhr et al. manipulated the genome of zebrafish embryos by placing the coding sequence for a fluorescent reporter under the control of JRS1 sequences from other species. The JRS1 region of all the vertebrates studied, including humans, induced expression in the jaw joint and surrounding cartilage, despite the enhancer sequence varying slightly between species. Further experiments provided additional details about the role of JRS1 in zebrafish, showing that zebrafish embryos genetically modified to lack a functional JRS1 enhancer expressed less *nxk3.2*, which caused their upper and lower jaw to transiently fuse (Figure 1).

Lampreys lack the JRS1 sequence and associated expression of *nxk3.2* in their first pharyngeal arch, which will go on to form the cartilage, bone and other structures of the jaw. However, other genes involved in jaw patterning are still expressed during lamprey development, suggesting that JRS1 appeared late in jaw joint evolution (Cerny et al., 2010). In particular, lamprey embryos activate several genes that control the identity of the upper and lower jaw in other vertebrates, the significance of which is an exciting subject for future studies.

There is growing recognition of how enhancers impact development and disease, but very few have been shown to have essential roles in vivo, or to contribute to morphological evolution. The work of Leyhr et al. has important implications for the basic understanding of jaw evolution, and could potentially help researchers identify the genetic causes underlying craniofacial defects.
